# Dr. William H. Pancoast

**Published:** 1897-02

**Authors:** 


Dr. William H. Pancoast, of Philadelphia, died at his residence, 1100 Walnut street, in that city, January 5, 1897, aged 63 years. The immediate cause of his death was pneumonia, although he had been somewhat enfeebled for a long period. Dr. Pancoast was a native of Philadelphia, where he was born October 16, 1835, and he was the son of Dr. Joseph Pancoast, the eminent anatomist, who for half a century occupied a foremost place in medical science in the United States. Dr. Pancoast, Jr., was educated at Haverford College, from which he received the degree of A. B. in 1853. He graduated from Jefferson Medical School with the degree of M. D. in 1856. After spending some years in the leading universities of London, Paris and Vienna, he returned to Philadelphia in 1859, when he was appointed one of the trustees of Charity Hospital and became a member of the surgical staff of that institution. In 1865, he was appointed demonstrator of anatomy at Jefferson, in which capacity he served with great credit for twelve years. Upon the resignation of his father, Dr. Joseph Pancoast, in 1874, William H. was appointed to fill the vacancy thus created in the chair of anatomy at Jefferson. He held this place until 1886, when he resigned and went abroad for a period of rest. Soon after his return he became president of the board of trustees of the medico-chirurgical hospital then lately established. He became professor of clinical and anatomical surgery in the medico-chirurgical college, which place he held until his death. He was a public-spirited citizen and a great lover of his native city. Foremost in all that pertained to art and science, he became a well-known figure at meetings of that character as well as an active participant in medical societies, local, state and national. He was a companion of the Military Order of the Loyal Legion. His funeral was largely attended by his professional colleagues, men of science, public officials and citizens.

